# Evaluation of In Vivo Adhesion Properties of New Generation Polyglactin, Oxidized Regenerated Cellulose and Chitosan-Based Meshes for Hernia Surgery

**DOI:** 10.7759/cureus.18755

**Published:** 2021-10-13

**Authors:** Mehmet Gulmez, Ali Aktekin, Fugen Aker, Vildan Sanko, Serdar Sezer

**Affiliations:** 1 Department of General Surgery, Acibadem Mehmet Ali Aydinlar University Atakent Hospital, Istanbul, TUR; 2 Department of General Surgery, Giresun University Faculty of Medicine, Giresun, TUR; 3 Department of Pathology, University of Health Sciences, Hamidiye Faculty of Medicine, Haydarpaşa Numune Health Application and Research Center, Istanbul, TUR; 4 Department of Chemistry, Gebze Technical University, Kocaeli, TUR; 5 Department of Pharmacology, Suleyman Demirel University Faculty of Medicine, Isparta, TUR

**Keywords:** composite mesh, polypropylene, hernia, oxidized regenerated cellulose, chitosan

## Abstract

Introduction

Composite meshes coated with anti-adhesive barriers have been developed by taking advantage of the robustness of polypropylene meshes for use in hernia repair. We aimed to evaluate the effects of composite meshes containing polyglactin, polycaprolactone, oxidized regenerated cellulose and chitosan on the adhesion formation.

Methods

Forty-two Sprague Dawley male rats were divided into six groups of seven rats according to the content of the meshes used. A defect was created on the right abdominal wall of the rats and an oval composite mesh of 2 cm in diameter was placed over the defect and fixed. The rats were sacrificed under anesthesia on the 7th postoperative day. Macroscopic and histopathological examination was performed and the incorporation of the mesh with the abdominal wall and the presence of intraabdominal adhesions were evaluated.

Results

When the macroscopic findings of the rats were evaluated, there was a statistically significant difference between the rat groups in terms of the distribution of peritoneal adhesion scores (p<0.05). There was no statistically significant difference between the rat groups in terms of the distribution of inflammation, fibrosis and macrophage levels (p>0.05).

Conclusion

It was evaluated that the development of intraabdominal adhesion and the strength of adhesion decreased when biocompatible adhesion barriers with anti-adhesive properties such as oxidized regenerated cellulose and chitosan were used in the structure of composite meshes used in hernia repair. Hemostatic and antibacterial properties of these substances are promising to create the ideal mesh.

## Introduction

Intra-abdominal adhesions are seen after abdominal operations and these adhesions cause complications such as chronic abdominal pain, small bowel obstruction, infertility in women, and cause iatrogenic bowel injury in secondary operations performed for adhesions [1,2].

For these reasons, patients are frequently admitted to hospitals, repeated hospitalizations may be required and patients often have to be operated. This situation brings with it a great financial burden. A method that prevents the formation of intra-abdominal adhesions will eliminate the subsequent operations and the financial burden it will bring.

Prosthetic meshes made up of different materials have been developed to prevent complications caused by intra-abdominal adhesions, but the ideal mesh has not been created yet [3]. Today, the most commonly used prosthetic mesh for hernia repair is polypropylene mesh. Because the polypropylene mesh causes an intense inflammatory response compared to other meshes and causes adhesion to the visceral organs, especially the small intestine, composite meshes containing anti-adhesive barrier, especially suitable for intraperitoneal use in laparoscopic hernia repair have been developed in order to prevent adhesion on the visceral side.

In this study, composite meshes including polyglactin, polycaprolactone, oxidized regenerated cellulose and chitosan were used, which accelerated tissue growth and had anti-adhesive activity. We aimed to evaluate the effects of these meshes which would accelerate tissue regeneration by preventing complications that might occur due to mesh use on adhesion formation.

## Materials and methods

This experimental study was initiated after it was approved by the Local Ethics Committee for Animal Experiments of Istanbul Medipol University (9/30/2015-E-2500). The animals were cared for according to the principles of the National Institutes of Health publication “Guide for Care and Use of Laboratory Animals”. Forty-two Sprague Dawley male rats were used in the study. The rats were given standard rat food and water during the experiment. All rats were kept separately in cages where 12 hours of light and dark environment were provided at room temperature.

The rats were divided into six groups of seven rats each: Control group; polypropylene mesh, group A; polycaprolactone coated polypropylene mesh, group B; polycaprolactone coated polypropylene mesh containing 20% oxidized regenerated cellulose, group C; polycaprolactone coated polypropylene mesh containing 40% oxidized regenerated cellulose, group D; polyglactin coated polypropylene mesh containing 30% chitosan, group E; polyglactin coated polypropylene mesh containing 10% chitosan.

Surgical procedure

Surgeries were performed under sterile conditions. Rats were administered 100 mg/kg Ketamine (Ketalar®) and 5 mg/kg Xylazine hydrochloride (Rompun®) intramuscularly, to provide long-term anesthesia and analgesia. Tail pinch and extremity withdrawal responses were examined to understand the depth of anesthesia. The abdominal hair was shaved and the anterior abdominal wall was stained with povidone-iodine. The abdomen was entered with a midline incision. In the right abdominal wall, a 1x1 cm area of parietal peritoneum and partial muscle was excised at a distance of 2 cm from the medial wall to the midline incision. An oval 2 cm diameter mesh was placed with a 6-0 polypropylene continuous suture, leaving the medial wall 1 cm away from the midline incision. The abdominal wall was closed with 4-0 polydioxanone and the skin with 5-0 polydioxanone. Flunixin (Flumexin®) 0.01 mg/kg was administered subcutaneously once for postoperative analgesia following wound closure.

The rats were sacrificed under anesthesia with Ketamine and Xylazine hydrochloride on the 7th postoperative day. Macroscopic examination was performed and the union of the mesh with the abdominal wall (incorporation) and the presence of intra-abdominal adhesions were evaluated and noted. Then, for histopathological evaluation, the specimens were numbered according to the groups and placed in 10% formaldehyde and sent for pathological examination.

Macroscopic evaluation of adhesion

The presence and degree of intra-abdominal adhesions were evaluated according to the scoring modified from Greca et al. [4,5] (Table 1). The type of adhesion was evaluated according to the classification recommended by Zühlke et al. [6] (Table 2). In addition, the union of the mesh with the parietal peritoneum (incorporation) was graded. While evaluating incorporation, the mesh surface was divided into four areas and each area was graded as 25%. 

**Table 1:** Criteria and scores for peritoneal adhesions modified from Greca et al. [4,5].

Scores	Classification
1	No adhesion
2	Omentum adhesion at suture zone
3	Omentum adhesion up to 50% of the mesh surface
4	Omentum adhesion more than 50% of the mesh surface
5	Visceral adhesion at suture zone
6	Visceral adhesion at mesh

**Table 2:** Characteristics of adhesion types according to Zühlke et al. [6].

Type	Characteristics
1	Filmy adhesion, easy to separate by blunt dissection
2	Stronger adhesion; blunt dissection possible, partly sharp dissection necessary; beginning of vascularization
3	Stronger adhesion; lysis possible by sharp dissection only; clear vascularization
4	Very strong adhesion; lysis possible by sharp dissection only; organs strongly attached with severe adhesions; damage of organs hardly preventable

Histopathological examination

The mesh was excised 2 cm from its upper border. The specimen was washed lightly with distilled water and then fixed with 10% buffered formalin. Samples were divided into segments, each containing the abdominal wall, mesh, and transition zone. Tissues were routinely followed up with an automatic tissue tracking device and placed in paraffin blocks. Samples were cut 4-5 micrometers, stained with hematoxylin and eosin and Picro Sirius red, and examined blindly by the pathologist under both light microscopy and polarized microscopy.

Under the light microscope, the presence of inflammation, fibroblast proliferation, angiogenesis, granulation tissue, fibrosis, giant cells, macrophages and edema were examined at 200 magnification and were classified as grade 0 (none), grade 1 (mild), grade 2 (moderate) or grade 3 (diffuse) using a scale. Scoring was performed at the end of this grading. The presence of neutrophils and giant cells as a foreign body reaction was expected to show the biological incompatibility of the mesh.

Statistical analysis of data

NCSS (Number Cruncher Statistical System) 2007 (Kaysville, Utah, USA) program was used for statistical analysis. While evaluating the study data, in addition to descriptive statistical methods (minimum, maximum, median, frequency, ratio), Kruskal Wallis Test was used for the comparison of quantitative data in three or more groups, and Mann Whitney U test was used for pairwise comparisons. Fisher Freeman Halton Test was used to compare qualitative data. Significance was evaluated at p<0.01 and p<0.05 levels.

## Results

**Figure 1:**
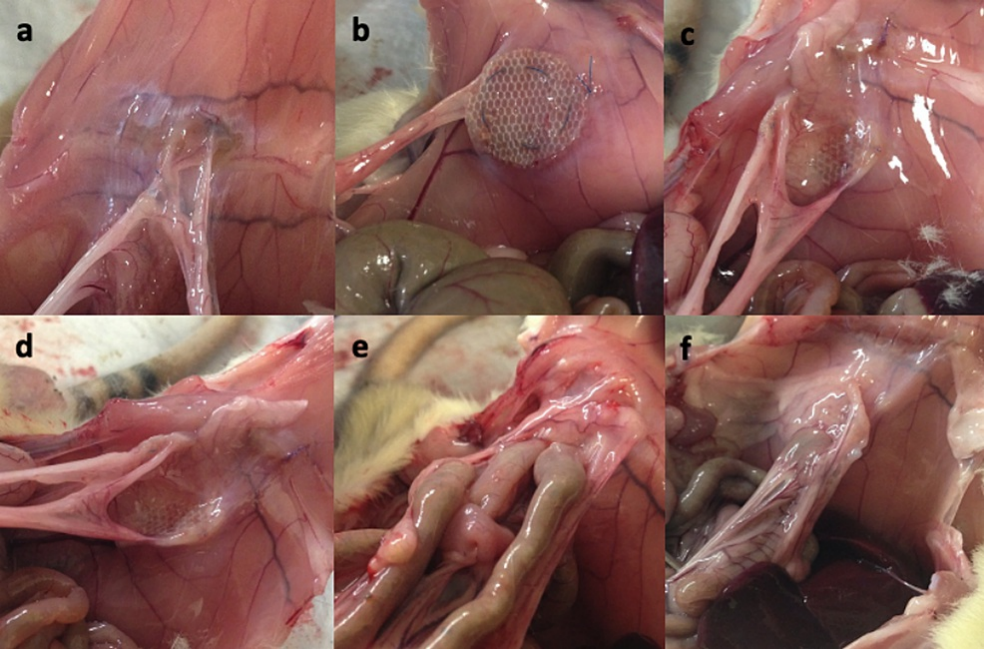
Macroscopic evaluation of peritoneal adhesion degrees after sacrification. (a) Omentum adhesion at suture line, (b, c) omentum adhesion up to 50% of the mesh surface, (d) omentum adhesion more than 50% of the mesh surface, (e, f) visceral adhesion at mesh.

When the macroscopic findings of the rats were evaluated, there was a statistically significant difference between the rat groups in terms of the distribution of peritoneal adhesion scores (p<0.05). The rate of visceral adhesion at the mesh, which was considered to be the strongest adhesion, was detected as 85.7% in the control group in which the polypropylene mesh was used, while this rate was 57.1% in group A in which only polycaprolactone coated polypropylene mesh was used and it was 28.6% when oxidized regenerated cellulose was added to this mesh. Visceral adhesion was detected to only one mesh in groups D and E in which polyglactin-coated polypropylene patches containing chitosan were used. The distribution of macroscopic findings according to the groups is shown in Table 3 and the peritoneal adhesion degrees that were evaluated macroscopically are shown in Figure 1.

**Table 3:** The distribution of macroscopic findings according to the groups. ^a^Fisher Freeman Halton test; ^b^Kruskal Wallis test; ^c^Min-Max(Median): minimum-maximum and median scores in each group according to scales. *p<0.05; **p<0.01. n(%): number and percentage of rats in each group.

		Control group	Group A	Group B	Group C	Group D	Group E	p
Peritoneal adhesion criteria and scores; n(%)	Omentum adhesion at suture zone	0 (0.0)	0 (0.0)	0 (0.0)	1 (14.3)	2 (28.6)	0 (0.0)	
	Omentum adhesion up to 50%	1 (14.3)	0 (0.0)	3 (42.9)	1 (14.3)	3 (42.9)	2 (28.6)	
	Omentum adhesion more than 50%	0 (0.0)	3 (42.9)	2 (28.6)	3 (42.9)	1 (14.3)	4 (57.1)	
	Visceral adhesion at mesh	6 (85.7)	4 (57.1)	2 (28.6)	2 (28.6)	1 (14.3)	1 (14.3)	
^c^Min-Max (Median)		3-6 (6)	4-6 (6)	3-6 (4)	2-6 (4)	2-6 (3)	3-6 (4)	^b^0.031*
Adhesion type; n(%)	Type 1	0 (0.0)	0 (0.0)	1 (14.3)	2 (28.6)	5 (71.4)	1 (14.3)	
	Type 2	0 (0.0)	0 (0.0)	3 (42.9)	1 (14.3)	2 (28.6)	3 (42.9)	
	Type 3	3 (42.9)	3 (42.9)	2 (28.6)	3 (42.9)	0 (0.0)	3 (42.9)	
	Type 4	4 (57.1)	4 (57.1)	1 (14.3)	1 (14.3)	0 (0.0)	0 (0.0)	
^c^Min-Max (Median)		3-4 (4)	3-4 (4)	1-4 (2)	1-4 (3)	1-2 (1)	1-3 (2)	^b^0.001**
Incorporation; n(%)	51%-75% of mesh surface	2 (28.6)	2 (28.6)	1 (14.3)	0 (0.0)	0 (0.0)	0 (0.0)	^a^0.375
	76%-100% of mesh surface	5 (71.4)	5 (71.4)	6 (85.7)	7 (100.0)	7 (100.0)	7 (100.0)	

When the distribution of types of adhesions was examined, a statistically significant difference was found between the groups. While the rate of type 4 adhesions was detected as 57.1% in the control group and group A rats, this rate was found to be significantly higher than the rats in the B, C, D and E groups (p<0.05). There was no statistically significant difference between the groups in terms of the incorporation grades of rats (p>0.05).

Fibroblast proliferation was moderately observed in 85.7% of the rats in the control group which was significantly higher than the A, B and C groups (p <0.05). However, moderate fibroblast proliferation was detected in all rats in groups D and E. While moderate angiogenesis was observed in 6 of the rats (85.7%) in the control group, similarly, moderate angiogenesis was detected in all rats in the group D and in 85.7% of the group E rats. Mild angiogenesis was detected in 5 (71.4%) of the rats in groups A and B and in all rats in group C. While 85.7% of the rats in the control group had moderate granulation, mild granulation was observed in most of the rats in groups A, B and C, and moderate granulation was detected in all rats in groups D and E. The distribution of histopathological findings according to the groups is shown in Table 4.

**Table 4:** The distribution of histopathological findings according to the groups. ^a^Fisher Freeman Halton test; ^b^Kruskal Wallis test; ^c^Min-Max(Median): minimum-maximum and median scores in each group according to scales. *p<0.05; **p<0.01. n(%): number and percentage of rats in each group.

		Control group	Group A	Group B	Group C	Group D	Group E	p
Inflammation; n(%)	Mild	2 (28.6)	4 (57.1)	6 (85.7)	6 (85.7)	5 (71.4)	6 (85.7)	^a^0.161
	Moderate	5 (71.4)	3 (42.9)	1 (14.3)	1 (14.3)	2 (28.6)	1 (14.3)	
Fibroblast proliferation; n(%)	Mild	1 (14.3)	5 (71.4)	6 (85.7)	5 (71.4)	0 (0.0)	0 (0.0)	^a^0.001**
	Moderate	6 (85.7)	2 (28.6)	1 (14.3)	2 (28.6)	7 (100.0)	7 (100.0)	
Angiogenesis; n(%)	Mild	1 (14.3)	5 (71.4)	5 (71.4)	7 (100.0)	0 (0.0)	0 (0.0)	
	Moderate	6 (85.7)	2 (28.6)	2 (28.6)	0 (0.0)	7 (100.0)	6 (85.7)	
	Diffuse	0 (0.0)	0 (0.0)	0 (0.0)	0 (0.0)	0 (0.0)	1 (14.3)	
^c^Min-Max (Median)		1-2 (2)	1-2 (1)	1-2 (1)	1-1 (1)	2-2 (2)	2-3 (2)	^b^0.001**
Granulation; n(%)	Mild	1 (14.3)	5 (71.4)	6 (85.7)	3 (42.9)	0 (0.0)	0 (0.0)	^a^0.001**
	Moderate	6 (85.7)	2 (28.6)	1 (14.3)	4 (57.1)	7 (100.0)	7 (100.0)	
Fibrosis; n(%)	Mild	7 (100.0)	7 (100.0)	5 (71.4)	6 (85.7)	7 (100.0)	7 (100.0)	^a^0.407
	Moderate	0 (0.0)	0 (0.0)	2 (28.6)	1 (14.3)	0 (0.0)	0 (0.0)	
Giant Cells; n(%)	Mild	5 (71.4)	1 (14.3)	7 (100.0)	7 (100.0)	0 (0.0)	2 (28.6)	
	Moderate	1 (14.3)	0 (0.0)	0 (0.0)	0 (0.0)	1 (14.3)	5 (71.4)	
	Diffuse	1 (14.3)	6 (85.7)	0 (0.0)	0 (0.0)	6 (85.7)	0 (0.0)	
^c^Min-Max (Median)		1-3 (1)	1-3 (3)	1-1 (1)	1-1 (1)	2-3 (3)	1-2 (2)	^b^0.001**
Macrophages; n(%)	Mild	4 (57.1)	7 (100.0)	6 (85.7)	7 (100.0)	7 (100.0)	7 (100.0)	^a^0.066
	Moderate	3 (42.9)	0 (0.0)	1 (14.3)	0 (0.0)	0 (0.0)	0 (0.0)	
Edema; n(%)	None	7 (100.0)	4 (57.1)	1 (14.3)	4 (57.1)	7 (100.0)	7 (100.0)	
	Mild	0 (0.0)	2 (28.6)	2 (28.6)	1 (14.3)	0 (0.0)	0 (0.0)	
	Moderate	0 (0.0)	1 (14.3)	4 (57.1)	1 (14.3)	0 (0.0)	0 (0.0)	
	Diffuse	0 (0.0)	0 (0.0)	0 (0.0)	1 (14.3)	0 (0.0)	0 (0.0)	
^c^Min-Max (Median)		0-0 (0)	0-2 (0)	0-2 (2)	0-3 (0)	0-0 (0)	0-0 (0)	^a^0.001**

There was no statistically significant difference between the groups in terms of the distribution of inflammation, fibrosis and macrophage levels of rats (p>0.05).

## Discussion

Abdominal adhesions are abnormal fibrotic bands between organ surfaces in the abdomen or between the walls of the abdominal cavity. In patients who have undergone abdominal or gynecological operations, intraabdominal adhesion formation is seen in 95% [7]. The main causes of adhesion formation are peritoneal trauma, ischemia and foreign bodies. Laparoscopic and minimally invasive techniques have been adopted to reduce the trauma that may occur during surgical intervention. Laparoscopic surgery is associated with less intraabdominal adhesion than classical open surgery, but postoperative adhesion still occurs in 37.7% of patients operated with laparoscopic methods [8]. Surgical technique is not sufficient to reduce postoperative adhesions and related complications. In order to prevent adhesions, besides choosing the surgical technique to minimize peritoneal damage, it is necessary to reduce the inflammatory response, provide inhibition of coagulation, stimulate fibrinolysis, and protect the surfaces that may cause adhesions [9].

Polypropylene mesh, which is the most widely used prosthetic material for hernia repair today, causes intense inflammatory response compared to other meshes and causes adhesion in visceral organs, especially small intestines. It is not suitable for intraperitoneal use due to complications such as fistula and small bowel obstruction. For this reason, composite meshes have been developed for use in intraperitoneal hernia repair. Bilayer composite meshes are preferred to prevent intraabdominal adhesion due to the strength and integration properties of permanent meshes such as polypropylene on the parietal peritoneum side and the intestinal protective property of the anti-adhesive barrier on the visceral side [10]. There are clinical studies showing that composite meshes are associated with shortened hospital stay, moderate complication rates, and low rates of infection and recurrence of hernia [11].

Today, the most widely used composite meshes around the world are polyglactin-based ones. Polyglactin is an absorbable and biocompatible polymer. It has proven its reliability and it is widely used in materials developed for tissue engineering and temporary implants such as surgical threads [12]. Materials such as polyurethane, polytetrafluoroethylene (PTFE), oxidized regenerated cellulose, polyethylene glycol, sodium hyalulose, carboxy methyl cellulose and collagen are used as anti-adhesives on the visceral surfaces of composite meshes [13]. Among these, oxidized regenerated cellulose used in Proceed® meshes has been reported to be superior to other materials in terms of anti-adhesive effects in experimental studies [13].

Aramayo et al. did not find a statistically significant difference between the groups in terms of the presence and degree of adhesion in an experimental study on 40 rabbits. A polypropylene mesh was used in one group, a mesh containing polypropylene and absorbable polygleocapron 25 (Ultrapro®) in other group, and a mesh containing polypropylene, polydiaxanone and oxidized regenerated cellulose (Proceed®) in another group [14]. In our study, no statistically significant difference was found between the control group in which the polypropylene mesh was used and the groups A, B, and C in which polypropylene and polycaprolactone meshes were used, in terms of peritoneal adhesion criteria and scoring. When oxidized regenerated cellulose was added as an adhesion barrier to the polycaprolactone coated polypropylene mesh (group B and C), the decrease in the degree of adhesion was not statistically significant compared to group A in which the mesh without adhesion barrier was used. When oxidized regenerated cellulose was added as an adhesion barrier to the polycaprolactone coated polypropylene mesh, it was evaluated that the adhesion rate of type 4 showing strong adhesion was low and that the oxidized regenerated cellulose caused a decrease in the adhesion strength.

Chitin, which is the most common polysaccharide-based biopolymer in the world after cellulose, is the main component of shellfish such as crab and shrimp, and is found in the skeleton of insects and the cell walls of fungi. Different molecules are obtained with the changes made in the structure of the chitin. The most important of these molecules is chitosan. The fact that chitosan has both antibacterial and hemostatic effects has made it attractive for use in the biomedical field [15,16]. Due to the fragile structure of chitosan and its limited use due to its inadequate mechanical properties, this problem can be overcome by forming composites together with other polymers.

When visceral organs come into contact with the polypropylene mesh, dense adhesions occur. In our study, in the control group in which polypropylene mesh was used, the rate of visceral adhesion to the mesh, which was the highest level of peritoneal adhesion score, was found to be higher than the B and C groups without reaching a statistical significance, but it was found to be significantly higher than the D and E groups. These findings showed that using the adhesion barrier together with the polypropylene mesh instead of using only the polypropylene mesh could reduce the adhesion macroscopically, and that the use of chitosan as the adhesion barrier significantly reduced the adhesion formation. In the experimental study by Altınel et al. in which polypropylene mesh, mesh containing polypropylene and absorbable polygleocapron 25 (Ultrapro®) and mesh created by adding chitosan to polypropylene and absorbable polygleocapron 25 (Ultrapro®) were used on rats; the groups were compared in terms of adhesion score and strength, and no significant difference was found between the adhesion scores of mesh groups and their chitosan-coated forms [17]. In another study, Paulo et al. observed the effects of chitosan on the formation of adhesion when combined with polypropylene mesh on rats in an experimental study using a polypropylene mesh and chitosan-coated polypropylene mesh, and stated that the amount of adhesion decreased when chitosan was added as an adhesion barrier in the mesh structure compared to the groups in which only the polypropylene mesh was used [18].

Utiyama et al. performed incisional hernia repair on rats and compared the histopathological characteristics of the groups (the group in which a mesh was not used, the group in which a polypropylene mesh was used and the group in which a mesh containing polypropylene and polygleocapron 25 [Ultrapro®] was used) in terms of inflammatory response. They stated that there was no difference between the groups in terms of fibrosis, macrophage, lymphocyte, neutrophil and giant cell counts, and granuloma [19]. Similarly, in our study, no statistically significant difference was found between the groups in terms of inflammation levels, distributions of fibrosis levels and macrophage levels.

There are some limitations of our study. At first, although the seven-day period was sufficient to evaluate the differences between the groups on adhesion formation, it was not possible to evaluate the long-term anti-adhesion efficacy of the meshes used. Also, considering the effectiveness of bleeding on adhesion formation, we think that when a material with hemostatic properties such as oxidized regenerated cellulose is added to the structure of the mesh, the hemostatic effect can be evaluated with different parameters. 

The model must be easily applicable and reproducible in surgical research and especially in vivo applications of innovations in biomedicine. Rats are one of the most suitable animals in terms of surface area in adhesion formation models. It is also known that the response to peritoneal trauma is similar to humans. In addition to all these, it is unclear how the degree of adhesions will make a difference in humans thus, the effects of these meshes on humans should be evaluated.

## Conclusions

Composite meshes with anti-adhesive barriers were developed for use in hernia repair and when placed intraperitoneally visceral surface of the mesh acts as a barrier for adhesion formation. This study showed that when adhesion barriers with biocompatible, anti-adhesion properties such as oxidized regenerated cellulose and chitosan are used in the structure of the composite meshes in hernia repair, the development of intra-abdominal adhesion and the strength of adhesion may decrease. Considering that these materials also have hemostatic and antibacterial properties, they can be promising to create the ideal mesh.
